# Sesquichloride of Iron in Rheumatism

**Published:** 1871-12

**Authors:** 


Sesquichloride of Iron as a Prophylactic in Acute Rheumatism.—Dr. Austin reports, in *The Practitioner* of September, twenty-nine cases of threatened acute rheumatism with this remedy. He says: "Whereas threatenings of gout could be very commonly dealt with in such a manner as to prevent the attack or render it trivial, the onset of acute rheumatism seemed never to be averted by drugs when once the prodromata had reached the stage which pretty frequently presented itself before me, viz., a more or less obscure aching of several joints, a yellow sallowness of face, with patches or streaks of dusky redness, blanket-like furring of tongue, an oily moisture of skin, a distinct though slight elevation both of pulse and temperature, and a certain anxiety of respiration. So far as the history of such patients could be traced, they were most invariably found to have developed the full symptoms of the acute disease, and very often (after once seeing them in the out-patient's room) one encountered them a few days later in the ward of the hospital.

Very different have been the results of treatment since I adopted the use of sesquichloride of iron from the first moment of such cases presenting themselves. During the past twelve months I have done this fully. Whenever a patient has presented himself with articular pain and slight fever that were plainly of the rheumatic, and not of the gouty type, he has been at once placed on thirty or forty minim doses of the tincture of the sesquichloride, from three to six of which, according to the severity of the symptoms, have been given in each twenty-four hours.

Since July, 1870, I have treated twenty-nine such patients, of whom thirteen had previously had one or more regular attacks of rheumatic fever, for the symptoms now referred to, with full doses of iron; and of these, seventeen have lost all pyrexia and spontaneous joint pain within the next three or four days.

Only three have, under my own eyes, developed the full acute disease, and been sent into the ward. Of the remaining nine, four disappeared altogether from my knowledge; the other five, though their symptoms were checked, remained in a state of what may be described as sub-acute rheumatism, during from ten to twenty days.

I can not help remarking with emphasis on the contradiction to old ideas which is involved in the effect of this iron treatment upon the furred tongue. Of course it becomes speedily blackened, but so far from the furring increasing, or the dryness and foul taste becoming more pronounced, what commonly happens is, that after a few days the epithelial coating falls off in patches, and the tongue soon cleans altogether."—*Med. Record.*

